# TRENDS IN HOUSEHOLD MATERIAL RESOURCES AND COGNITIVE HEALTH IN A LONGITUDINAL COHORT STUDY OF AGING IN SOUTH AFRICA

**DOI:** 10.1093/geroni/igac059.677

**Published:** 2022-12-20

**Authors:** Lindsay Kobayashi, Chodziwadziwa Kabudula, Mohammed Kabeto, Xuexin Yu, Stephen Tollman, Kathleen Kahn, Lisa Berkman, Molly Rosenberg

**Affiliations:** University of Michigan, Ann Arbor, Michigan, United States; MRC/Wits Rural Public Health and Health Transitions Research Unit (Agincourt), University of the Witwatersrand, Johannesburg, Gauteng, South Africa; University of Michigan, Ann Arbor, Michigan, United States; University of Michigan, Ann Arbor, Michigan, United States; MRC/Wits Rural Public Health and Health Transitions Research Unit (Agincourt), University of the Witwatersrand, Johannesburg, Gauteng, South Africa; MRC/Wits Rural Public Health and Health Transitions Research Unit (Agincourt), University of the Witwatersrand, Johannesburg, Gauteng, South Africa; Harvard Center for Population and Development Studies, Harvard T.H. Chan School of Public Health, Cambridge, Massachusetts, United States; Department of Epidemiology and Biostatistics, Indiana University School of Public Health-Bloomington, Bloomington, Indiana, United States

## Abstract

Material resources that affect daily living conditions may be salient for cognitive aging in low-income settings, but evidence is limited on this topic. We investigated relationships between long-term trends in household material resources and subsequent cognitive function among 4,580 adults aged ≥40 in a population-representative cohort in Agincourt sub-district, South Africa, from 2001-2015. Household material resources (dwelling materials, water, sanitation, sources of power, modern amenities, and livestock) were assessed biennially from 2001-2013. We evaluated mean resources, volatility in resources, and change in resources over this period in relation to cognitive function in 2014/2015. Higher mean household resources and larger improvements over time in resources were positively associated with subsequent cognitive function, independent of confounders. Findings were largely driven by modern amenities for food preparation, transportation, and communication outside of the household. Access to these amenities may support cognitive aging through boosting nutrition and cognitive reserve and should be investigated further.

